# Emotional distress, stress, anxiety, and the impact of the COVID-19 pandemic on early- to mid-career women in healthcare sciences research

**DOI:** 10.1017/cts.2022.417

**Published:** 2022-06-13

**Authors:** Noor Bittar, Andrea Cohee, Surya Sruthi Bhamidipalli, April Savoy, Heba M. Ismail

**Affiliations:** 1 Department of Pediatrics, Indiana University School of Medicine, Indianapolis, IN, USA; 2 Department of Community and Health Systems, Indiana University School of Nursing, Indianapolis, IN, USA; 3 Department of Biostatistics and Health Data Sciences, Indiana University School of Medicine & Richard M. Fairbanks School of Public Health, Indianapolis, IN, USA; 4 Center for Health Information and Communication, U.S. Department of Veterans Affairs, Veterans Health Administration, Health Services Research and Development Service CIN 13-416, Richard L. Roudebush VA Medical Center, Indianapolis, IN, USA; 5 Regenstrief Institute, Inc., Indianapolis, IN, USA; 6 Indiana University Center for Health Services and Outcomes Research, Indianapolis, IN, USA; 7 Purdue School of Engineering and Technology, Indiana University-Purdue University, Indianapolis, IN, USA

**Keywords:** Stress, anxiety, women, academia

## Abstract

**Objectives:**

The main objective of this study was to report stress and anxiety levels during the COVID-19 pandemic on early- to mid-career women researchers in healthcare sciences research and determine the associated factors.

**Methods:**

A 50-item self-administered internet questionnaire was developed using a mix of Likert-type scales and open-ended response questions. The survey was distributed June 10–August 3, 2020. Anxiety and stress as well as personal/family demands were assessed through validated measures (Patient Reported Outcomes Measurement Information System [PROMIS]-Anxiety Short Form and Perceived Stress Scale [PSS]) and open-ended responses.

**Results:**

One hundred and fifty-one early-career women in healthcare sciences research completed the survey; mean respondent age was 37.3 ± 5.2 years; and all had a college degree or higher, 50.3% holding a PhD and 35.8% MD. Race and ethnicity were reported in 128; the majority were White (74.0%). One-third (31.2%) reported being “very much” concerned about reaching their research productivity goals and 30.1% were “very much” concerned about academic promotion and tenure. Fifty percent reported a “moderate” PROMIS anxiety score and 72.1% reported a “moderate” PSS score. For the open-ended responses, 65.6% reported a worry about their professional goals because of the COVID-19 pandemic. Major concerns revolved around finances, childcare, and job security.

**Conclusions:**

Throughout the pandemic, early- and mid-career women in healthcare sciences research have reported moderate to high overall stress, anxiety, and worries. These concerns appear related to household settings, additional responsibilities, financial concerns, and reduced research productivity. Institutions and funding agencies should take these concerns into consideration and offer support.

## Introduction

Early- and mid-career women researchers in healthcare sciences research often face multiple stressors and challenges while balancing their personal and professional roles [1]. It is well documented that women in academia struggle with male-dominated institutional cultures, competing family responsibilities, and biases in recruitment, research support, and publication [2]. Unfortunately, with the COVID-19 pandemic, these challenges have been amplified. In particular, those who are engaged in research face delays in their ongoing studies from stay-at-home orders and shutdowns.

Compared to men, many women working from home during the pandemic have reported spending more time on childcare [3]. While most careers have been negatively impacted, early-career women in healthcare sciences research have been noted to be at particularly high risk for experiencing a negative impact [4]. Furthermore, those who hold both clinical and academic positions have been either redeployed or asked to perform additional administrative duties, diverting their attention from their academic scholarly work [5,6]. These challenges have led to a significant decrease in productivity among researchers, with 85% of faculty members in one study stating that they expect to decline, postpone, or cancel publishing or a research commitment due to COVID-19 [7].

Therefore, it is critical to understand the extent of stress and anxiety on early- and mid-career women in healthcare sciences research and potential sources that could be intervened upon. In this study, we explored the impact of the pandemic on this population. We hypothesized that early- and mid-career women healthcare sciences researchers would report greater stress and anxiety related to the pandemic along with increased responsibility and reduced productivity. We aimed to 1) report stress and anxiety levels using validated measures during the early period of the pandemic and 2) determine factors associated with increased stress and anxiety among these women during the pandemic. We anticipate that these findings will help research institutions and funding agencies in drafting policies to support this population following future pandemics and other natural disasters.

## Methods

### Study Design

This cross-sectional study was submitted, reviewed, and considered an Institutional Review Board exempt study by the Indiana University IRB (IRB Protocol #2006034715). A 50-item questionnaire was created through Qualtrics (Qualtrics, Provo, UT) and distributed nationwide from June 10 to August 3, 2020. The survey was distributed via social media platforms such as Facebook and Twitter, institutional listservs, newsletters, and emails at professional organization at large public and private universities, the American Medical Women’s Association, as well as by word-of-mouth. No incentives were provided for study participation.

### Eligibility Criteria

A priori inclusion and exclusion criteria were established to identify participants eligible for this study. Inclusion criteria were individuals who identified as women working in health sciences research, at least 15% of their professional time and effort spent on research, able to read and understand English, and in the early to mid-stages of their career (research fellows to associate professor level). Those who did not meet any of those inclusion criteria were discontinued from participation in the survey using a gated question method.

### Survey Instrument

The survey instrument (Supplemental survey file) combined demographic and career status questions with specific questions regarding the impact of COVID-19 on research, professional support, communication, finances, productivity, childcare, and feelings. Additionally, respondents were asked about COVID-19’s impact on their stress and anxiety using validated measures (see *Measures*). Some questions were asked in the form of “yes/no” or “select all that apply” questions, while a majority asked participants to rate their responses on a five-point Likert scale. The questionnaire also included six open-ended questions. During the design process for this questionnaire, the study team consulted with the Center for Survey Research at Indiana University. Several revisions were made, and the survey was pretested and piloted among the investigators with follow-up recommendations that lead to the final version.

### Measures

#### Demographics

The following demographic data were recorded from survey responses: age, race, ethnicity, degree(s), profession, specialty of practice (if applicable), career track, and average income. Additional data collected included number of years in current position, number of years in research, percent of time spent in clinical, teaching, and administrative duties, as well as type of institution (public university, hospital). In addition, participants were asked about their marital status and household members including children, seniors, and members with special needs.

#### Stress

Stress was measured using the Perceived Stress Scale (PSS), a 10-question validated measure which asks about the frequency of specific thoughts and feelings in the last month. These included how often respondents felt on top of things, felt able to control irritations in one’s life, and felt confident in their ability to handle personal problems, et cetera. Responses were scaled on a 5-point Likert scale (“Never,” “Almost Never,” “Sometimes,” “Fairly Often,” “Very Often”) and were summed to give a result between 0 and 40, with scores of 0–13 indicating low stress, 14–26 moderate stress, and 27–40 high stress [8].

#### Anxiety

Anxiety was measured using the PROMIS Anxiety Short Form, a validated measure of 8 questions evaluating participant feelings over the prior 7 days, specifically assessing how often respondents felt nervous, uneasy, fearful, anxious, et cetera. Questions were asked on a 5-point Likert scale (“Never,” “Rarely,” “Sometimes,” “Often,” “Always”). Responses were summed and scored, converted into T-scores and categorized as “none to slight” (0–55), “mild” (55–59.9), “moderate” (60–69.9), and “severe” (>70) [9].

#### COVID and Self-Efficacy

Questions also asked about respondents’ confidence in taking care of themselves and household members as well as how much any emotional distress caused by COVID-19 had interfered with self-care and household members’ care. Responses were measured on a 5-point Likert scale (“Not At All,” “A Little,” “Somewhat,” “Quite a Bit,” “Very Much”).

#### COVID and Feelings

Eight questions asked about respondents’ feelings secondary to the pandemic. Examples included feeling angry, depressed, anxious, etc. Responses were measured on a 5-point Likert scale (“Not At All,” “A Little,” “Somewhat,” “Quite a Bit,” “Very Much”). Internal agreement between these 8 questions was analyzed using Cronbach’s alpha test (α > 0.8).

#### Open-Ended Questions and Responses

The survey also included open-ended questions. These questions allowed respondents to provide further detail regarding how the COVID-19 pandemic had affected their personal and professional lives. Questions ranged from the most difficult aspects of the pandemic in terms of personal and professional life and worries due to the COVID-19 pandemic to coping mechanisms throughout the pandemic. Individual responses to open-ended questions were quantitatively categorized based on similar responses and grouped according to common themes by two study team members (NB and HMI) and cross-checked.

#### Data Synthesis

A total of 185 survey responses were collected from this study. One hundred and fifty-one responses met inclusion criteria and 34 were excluded due to being incomplete. All 34 incomplete surveys only answered the first question regarding gender and did not proceed with the remainder of the survey.

Data were summarized for demographic variables including age, race, highest degrees, number of years conducting research, current position, length of time in current position, place of work, and by categories of anxiety and perceived stress. Data were also summarized for variables regarding COVID-19 and research, finances, productivity, and diagnosis. The one-way ANOVA test was used to test for the differences among the groups of PROMIS anxiety and PSS stress scores for continuous variables. Chi-square test was used to test for the differences/associations for categorical variables. Kruskal-Wallis test was used to determine the correlation between the continuous variables and the categories of anxiety and stress.

Variables that were significant in Chi-square analyses with the PROMIS anxiety and PSS stress scales were then entered into a logistic regression analysis to determine the effect of these variables on prediction of stress and anxiety. All the tests were run at the level of 5% significance (two-sided alpha). The statistical software SAS 94. (NC, Cary) was used for the statistical analysis of the data.

## Results

### Demographics

One hundred fifty-one women in healthcare sciences research completed the survey. The demographic data for participants are shown in Table 1. The mean respondent age was 37.3 ± 5.2 years. All respondents had a college degree or higher, with 50.3% holding a PhD, 35.8% an MD, and 11.3% held a combined MD/PhD. Fifty-two percent were classified as Scientists and 47.7% were Physician Scientists. The majority (80.3%) held an Assistant Professor position. At the time of the survey, 72.9% of these women had been in their respective positions for 1–5 years and had been conducting research between 5 and 10 years (43.1%), with 59.3% noted working at a large public university.

**Table 1.** Demographics and characteristics

	37.29 ± 5.2 years	
Mean Age	Number	%
Race (*n* = 128)		
White	N = 94	74.0
Black/AA	N = 5	3.9
Hispanic/Latino	N = 6	4.7
Asian	N = 14	11.0
Multiracial	N = 8	6.3
Unspecified	N = 1	0.0
Highest Degree (N = 151)		
MD	N = 54	35.8
PhD	N = 76	50.3
MD, PhD	N = 17	11.3
Masters or less (MPH, MPA, Bachelors)	N = 4	2.7
Length of time conducting research (N = 151)		
< 6 months	N = 3	2.0
1–5 years	N = 50	33.1
5–10 years	N = 65	43.1
15–20 years	N = 28	18.5
> 20 years	N = 5	3.3
Current Position (N = 151)		
Associate Professor	N = 16	10.6
Assistant Professor/Lecturer/Instructor	N = 99	65.6
Post-doctoral/Clinical Fellow	N = 7	4.6
Others (Federal Researcher, Admin. Position, Technician, Research Manager)	N = 29	19.2
Scientist or Physician (N = 151)		
Scientist	N = 79	52.3
Physician Scientist	N = 72	47.7
Time in current position (N = 151)		
< 6 months	N = 8	5.3
1–5 years	N = 110	72.9
5–10 years	N = 30	19.9
15–20 years	N = 3	2.0
> 20 years	N = 0	0.0
Place of work (N = 150)		
Federal	N = 5	3.3
Research institute	N = 2	1.3
Large public university	N = 89	59.3
Large private university	N = 26	17.3
Small private university	N = 2	1.3
State university	N = 21	14.0
Hospital and medical center (academic/non-academic)	N = 5	3.3
Total annual family income (N = 132)		
< 99,000	N = 17	12.9
99,000–175,000	N = 56	42.4
> 175,000	N = 59	44.7
Missing	N = 19	

Of the 151 respondents, 128 reported their race/ethnicity, with a majority identifying as White (74.0%). Out of 132 women who reported their current marital/family status and annual family income, 65.9% were married with children and 44.7% had an annual family income of > $175,000, Table 1.

### Measures of Stress and Anxiety

Results indicated, for overall levels of stress measured using the Cohen’s PSS scale, that 50.0% of participants experienced at least a moderate level of stress, followed by 22.2% with severe levels of stress. Overall levels of anxiety measured using the PROMIS short-form questionnaire showcased 72.1% of participants reported moderate levels of anxiety and 19.4% high anxiety, Table 2. Demographic characteristics were not associated with stress and anxiety in this study. Current position and time in current position were not associated with stress or anxiety levels.

**Table 2.** Responses to patient reported outcomes measurement information system (PROMIS)-anxiety short form and Perceived Stress Scale (PSS)

	Number	Frequency
PROMIS anxiety scale [N = 126]		
None to slight	20	15.9
Mild	15	11.9
Moderate	63	50.0
Severe	28	22.2
PSS stress scale [N = 129]		
Low	11	8.5
Moderate	93	72.1
High	25	19.4

### COVID-19, Feelings and Baseline Comparison

Participants were asked to rank the pandemic’s impact on their personal well-being through various scaled questions assessing feelings of anxiety, fear, anger, and depression. While there was a varied distribution in responses, a majority stated that there was at least “Somewhat” or “Quite a Bit” of a disruption in their life and mental and emotional well-being as a result of the pandemic. The mean ± SD COVID-19 “feelings scale” score was 3.5 ± 0.7, which was highly reliable with a Cronbach’s alpha of 0.85. Negative feelings toward COVID-19 showed significant positive associations to both PSS and PROMIS anxiety scores and the degrees of stress and anxiety (*P* < 0.01 for both), with higher scores associated with both moderate and severe anxiety and stress, supplemental table 1.

### Baseline Stress and Anxiety

When participants were asked to rank their levels of anxiety during the COVID-19 pandemic in comparison to before the pandemic, a majority (58.3%) of participants scored levels of anxiety during the pandemic as “More than Before,” while 26.5% scored “Much more than before.” Similarly, 51.5% reported a stress level “More than Before” and 33.3% reported a stress level “Much more than before” the pandemic, supplemental table 1.

### Measures of Self-Efficacy

Thirty-two percent of respondents felt that COVID-19 interfered “A Little” with their ability to take care of themselves. When adding burdens of social distancing to their self-care, a large percentage (36.9%) stated that they felt “Quite a Bit” confident. Of the 32 women who answered how their COVID-19 related emotional distress interfered with the management of their household with special needs, 31.3% stated there was “Somewhat” of an interference. Lastly, 53.8% of 26 women answered that they were “Somewhat” confident in managing household members with special needs, Table 3. We did not observe associations in responses to the question “How confident are you that you can take care of yourself with the added burden of social distancing?” and PROMIS anxiety scores (X^2^ = 13.9, *P* = 0.31) and the question “How confident do you feel that you can manage your household members with special needs at home?” and the PSS stress scores (X^2^ = 19.8, *P* = 0.07). However, all other associations were statistically significant (*P* < 0.05), supplemental table 2.

**Table 3.** Participant responses to self-efficacy questions regarding confidence and distress during the COVID-19 pandemic

COVID-19 and self-efficacy	Not at all	A little	Somewhat	Quite a bit	Very much
How confident are you that you can take care of yourself with the added burden of social distancing? N = 130	2.31% (*n* = 3)	7.69% (*n* = 10)	20% (*n* = 26)	36.9% (*n* = 48)	33.1% (*n* = 43)
How much does emotional distress caused by COVID-19 interfere with taking care of yourself? N = 130	13.1% (*n* = 17)	32.3% (*n* = 42)	30.8% (*n* = 40)	16.9% (*n* = 22)	6.9% (*n* = 9)
How much does emotional distress caused by COVID-19 interfere with the management of household members with special needs? N = 32	15.6% (*n* = 5)	15.6% (*n* = 5)	31.3% (*n* = 10)	25.0% (*n* = 8)	12.5% (*n* = 4)
How confident do you feel that you can manage your household members with special needs at home? N = 26	3.9% (*n* = 1)	3.85% (*n* = 1)	53.9% (*n* = 14)	26.9% (*n* = 7)	11.5% (*n* = 3)

### Impact on Research and Institutional Support

While many participants who stated lab closures or research projects were on hold due to the COVID-19 pandemic, there were no significant correlations to stress and anxiety levels among participants. The same was true for participants who stated that they had taken on more responsibilities due to the pandemic. However, for those who stated the highest average amount of clinical effort, there were negative associations with anxiety levels as measured by PROMIS scores (*P* = 0.05) in the whole group, but no associations with stress levels. To further explore this negative association with clinical effort, we assessed stress and anxiety levels by percent effort among the two major subgroups, scientists and physician scientists. There was a significant yet modest negative correlation between clinical effort and each of the stress (ρ = −0.31, *P* = 0.01) and anxiety levels in scientists (ρ = −0.28, *P* = 0.02). There was a similar modest negative trend, but this was not statistically significant for physician scientists. There was also an interesting positive correlation between administrative work and stress among scientists (ρ = 0.39, *P* < 0.01), Table 4.

**Table 4.** Showing coefficient correlations (ρ) and significance levels for associations between each of anxiety and stress levels by percent effort work allocation for physician and physician scientists

Variable	Clinical ρ (*P*-value)	Teaching ρ (*P*-value)	Admin ρ (*P*-value)	Research ρ (P-value)
**Anxiety:**				
Scientists	−0.31 (0.01)	0.02 (0.90)	0.21 (0.09)	0.21 (0.09)
Physician scientists	−0.14 (0.30)	0.01 (0.94)	0.11 (0.40)	0.09 (0.50)
Differences^*^ Scientists vs Physician scientists	−0.93 (0.35)			
Stress				
Scientists	−0.28 (0.02)	0.13 (0.30)	0.39 (<0.01)	0.04 (0.75)
Physician scientists	−0.01 (0.92)	−0.03 (0.81)	0.13 (0.32)	−0.03 (0.85)
Differences^*^ Scientists vs Physician scientists	−1.45 (0.15)		1.40 (0.16)	

* Difference between the correlations of scientists and physician scientists were only tested when at least one correlation is statistically significant.

When reporting COVID-19-related concerns with research goals and productivity, 28.9% reported that they were “quite a bit” concerned and 45.9% were “very much” concerned. Most participants stated that their research productivity decreased with either increased or decreased work hours. Of those who reported “very much” concerned, 29.0% (*n* = 18) had severe anxiety and 27.4% (*n* = 17) had high stress levels. Of the participants who stated their productivity decreased while their work hours were reduced, the majority had moderate to high anxiety and stress levels, comprising 64.0% of those in the high stress category and 64.3% in the severe anxiety category, Table 5. Additional concerns of promotion/tenure, current and future funding, or salary cuts and furloughs were stated; however, there were no significant associations with levels of stress or anxiety.

**Table 5.** Productivity, work hours, and perceived anxiety and stress levels as measured using the patient reported outcomes measurement information system (PROMIS)-anxiety short form and Perceived Stress Scale (PSS)

			PROMIS-Anxiety									COHEN’s PSS-stress						
Variable	n/mean	%/SD	None to slight (*n* = 20)		Mild (*n* = 15)		Moderate (*n* = 63)		Severe (*n* = 28)		*P*-value	Low (N = 11)		Moderate (N = 93)		High (N = 25)		*P*-value
Due to the effects of the COVID-19 Pandemic			N	%	N	%	N	%	N	%	0.12	N	%	N	%	N	%	0.08
Productivity increased	4	3	3	15	0	0	1	1.6	0	0		2	18.2	2	2.2	0	0	
Productivity reduced	30	22.9	5	25	3	20	17	27.4	5	17.9		1	9.1	21	22.8	8	32	
Work hours increased	7	5.3	2	10	0	0	2	3.2	3	10.7		2	18.2	3	3.3	1	4	
Productivity reduced, work hours increased	12	9.2	1	5	3	20	7	11.3	0	0		2	18.2	9	9.8	0	0	
Productivity increased, work hours increased	3	2.3	0	0	0	0	2	3.2	1	3.6		0	0	3	3.3	0	0	
Productivity increased and/or work hours reduced	3	2.3	0	0	0	0	0	0	2	7.1		0	0	3	3.3	0	0	
Productivity reduced, work hrs reduced	72	55	9	45	9	60	33	53.2	17	60.7		4	36.4	51	55.4	16	64	

About twenty percent reported no-to-little support from either their institution or department, while the majority felt supported “Quite a bit” by their institution (35.5%) or department (35.9%), respectively.

### Impact on Finances and Responsibilities

The majority of respondents said there were no salary cuts or furloughs and that they had not experienced financial difficulty at the time of the survey. There was a significant difference in stress levels by total family income (*P* = 0.02), but not in anxiety levels. The majority (47.3%) of those in the moderate stress level as measured by PSS were in the mid-range income level of 99,000–175,000. Interestingly, while many mentioned childcare obligations as factors associated with their stress and anxiety levels in the open-ended questions, there were no significant differences among participants’ levels of stress and anxiety when reporting school-age children in their household, responsibility level, and/or being in charge of e-learning, Table 6. Specifically, 66.7% (*n* = 88/130) reported only children and adults in their household (but not seniors) and there were no significant associations seen with levels of stress or anxiety (*P* = 0.1 and 0.48, respectively). Similarly, in looking at the age of the children in the household, there were no associations with either stress or anxiety scales (*P* = 0.26 and *P* = 0.52, respectively). No significant association with stress and anxiety scores appeared with other COVID-19 and financial questions within the survey.

**Table 6.** COVID-19, finances and responsibilities’ responses

Questions [N = 132]	Number	Frequency
Have there been any salary cuts or furloughs to you, your staff, or anyone in your research team?		
Yes	21	15.9
No	111	84.1
Have you been asked to redeploy to clinical duties or provide information on willingness to redeploy?		
Yes	51	38.6
No	41	31.1
N/A	40	30.3
Have you experienced financial difficulty related to COVID-19?		
Yes	18	13.6
No	114	86.4
What is your current marital/family status?		
Married without children	23	17.4
Married with children	87	65.9
Single without children	18	13.6
Single with children	4	3.0
Missing	19	
Who lives in your household?		
Adults	39	29.6
Adults, Children	88	66.7
Adults, Children, Seniors (> 65 Years Old)	3	2.3
Missing	21	
Children in the household [N = 91]		
Children < 5 Years Old	32	35.2
Children < 5 Years Old, Children Between 5 and 12 Years	30	33.0
Children < 5 Years Old, Children Between 5 and 12 Years, Teenagers < 18 Years Old, Children with Special Needs	3	3.3
Children Between 5 and 12 Years and Teenagers	26	28.6
Are/were children receiving e-learning at home during the pandemic [N = 91]?		
Yes	67	73.6
No	24	26.4
Are/were you in charge of schooling and e-learning? [N = 67]?		
Yes, full time	27	40.3
Yes, part time	35	52.2
No	5	7.5
Due to the effects of the COVID-19 pandemic, the responsibility of childcare (or adult/senior care) has now		
Fallen mostly on me	28	30.1
Is currently equally shared with others	32	34.4
Is currently unequally share with others (others do most of it)	6	6.5
Is currently unequally shared with others (I do most of it)	27	29.0

### Impact of COVID-19 Diagnosis

Regarding whether participants or someone they knew had been diagnosed with COVID-19, the majority (54.5%, *n* = 72/132) said “No” with 86.4% (*n* = 114/132) of them reporting not currently caring for any patients with COVID-19 in their practice.

Interestingly, those who responded yes to each of the questions related to “knowing or having COVID-19” or “caring for someone diagnosed” in their practice also showed moderate to high levels of anxiety with a significant positive association with PROMIS Anxiety scores (*P* = 0.03 and *P* = 0.02, respectively). There were no associations seen with stress levels, and there were no associations seen between active or passive COVID-19-related information seeking behaviors and anxiety or stress levels.

### Regression Models

**Fig. 1.**
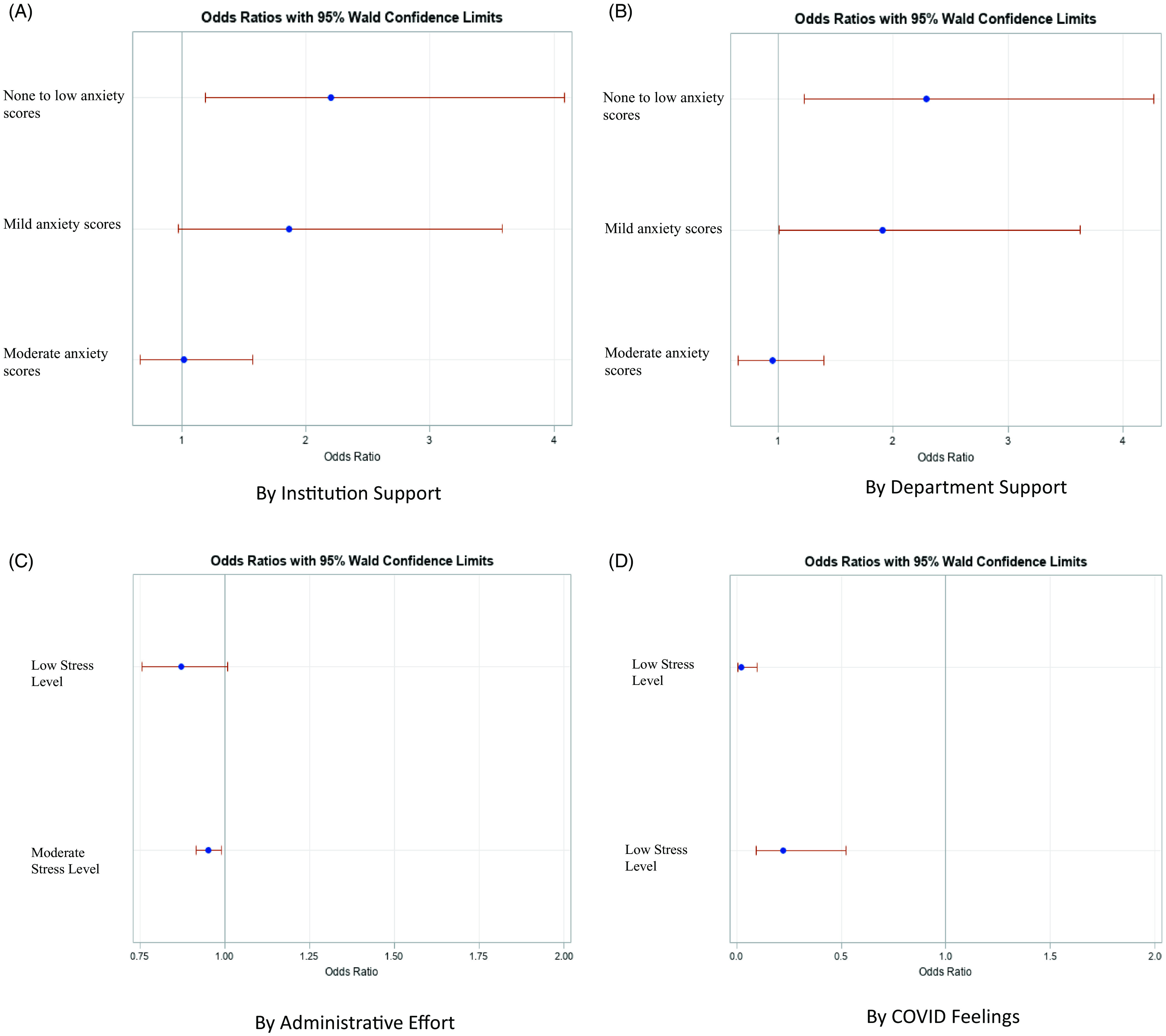
Logistic regression analysis for anxiety by A. Institutional support and by B. Department support. Logistic regression analysis for stress by C. Administrative effort and by D. COVID feelings.

With the reference group being the “severe” PROMIS anxiety scores, the odds of having low anxiety scores were greater if the participant felt supported by their institution (none to low anxiety scores OR = 2.2, CI 1.19, 4.08, *P* = 0.01) or by their department (none to low anxiety scores OR = 2.29, CI 1.23, 4.26, *P* = 0.01; mild anxiety scores OR = 1.91, CI 1.01, 3.62, *P* = 0.04), Fig. 1 A-B. Whereas the odds of increased time and percent effort spent on administrative tasks was lower in the “moderate” stress-level group compared to the “high” stress-level group as measured by PSS (OR = 0.95, CI 0.91–0.99, p-value 0.02), Fig. 1 C.

### Additional Open-Ended Questions

#### Career impacts

A large portion of the sample identified the effect on their career (*n* = 74/131; 56.5%), including reduced research work hours and productivity (*n* = 83; 63.4%). Reasons for reduced productivity included lack of focus and distraction (21.1%), stress (17.5%), and work hours being reduced (14.7%).

#### Worries

The majority (65.6%) of women reported a worry about their professional goals as a result of the COVID-19 pandemic. Major concerns revolved around finances, childcare, and job security. Financial status was addressed either on a personal level within their household or professional level due to grant cuts and grant deadlines not being met. Sixty-eight percent reported on their biggest professional concerns, with maintaining productivity and career impacts at the top of these professional concerns, supplemental table 3.

#### Challenges

A majority of respondents (*n* = 97, 64.2%) shared the most challenging aspects of managing their personal and professional lives during the COVID-19 pandemic, with additional responsibilities (childcare, added clinical hours) encompassing 29.0% of responses.

Childcare was a major theme among participants. In fact, 91 stated that they had children under 18 years of age in their households. Of those with children, 35.2% were younger than 5 years old. Seventy-four percent (*n* = 67/91) of children were receiving e-learning and only 67 responded to whether they were in charge of e-learning with 40.3% of participants reporting that they oversaw full-time learning (*n* = 27/67).

#### Coping Mechanisms

We also inquired about participants’ coping mechanisms for stress and anxiety. The majority (*n* = 103, 68.2%) responded to the question on whether they have someone to talk to about their concerns with 91 (88.3%) of women saying that they do have someone to speak with in some capacity. This encompassed 73.6% mentioning friends (colleagues, friends, peers), 38.5% (*n* = 35) a mentor, or supervisor and 15.4% (*n* = 14) mentioning family members (family, relatives, spouse). Other outlets included social media and online platforms (4.4%), therapists (3.3%), or an unspecified source (2.2%). A small number (*n* = 13) of respondents mentioned having someone to speak to about their concerns but not being able to reach out to them due to lack of time or believing they would not understand their concerns. Further, 11 other respondents said they did not have someone to voice concerns to.

The majority of respondents (*n* = 96, 63.6 %) reported on how they were coping with the stress associated with the pandemic. Coping mechanisms included physical activity (55 respondents), spending time with and talking with family and friends (41 respondents), optimism and leisure activities (*n* = 22), keeping up with a routine (*n* = 18), and eating and drinking alcohol (*n* = 20). Other less frequent coping strategies included limited social media use and therapeutic interventions such as meditation or therapy (*n* = 3). A small number (*n* = 5) of respondents stated they were not coping well or were overwhelmed with worry and childcare.

## Discussion

This study aimed to provide insight into the impact of the COVID-19 pandemic on women in healthcare sciences research in an effort to inform more equitable institutional policies in the field of science. Our results show that the majority had moderate anxiety and stress levels and about 20% had high levels. About a third were “very much” concerned about reaching their research productivity goals, academic promotion, and tenure. About two-thirds reported a worry about their professional goals as a result of the COVID-19 pandemic and major concerns revolved around finances, childcare, and job security. The majority listed their coping mechanisms, yet a handful reported they were not coping well.

Here, we focused specifically on the increased level of stress and anxiety that women in healthcare sciences research have encountered as a result of their professional obligations becoming further intertwined with family and other personal responsibilities. These problems amplified due to budget cuts, furloughs, redeployment, additional family and professional responsibilities, and concerns for long-term career impacts, consistent with what others have reported [4]. Women in academia and healthcare sciences research are challenged regularly with daily stressors, without the addition of a pandemic’s implications on their personal and professional well-being. Our predictions were supported in that women in healthcare sciences research reported higher levels of stress and anxiety as a result of the pandemic compared to prior to the pandemic.

This work highlights the issues women in healthcare sciences research settings encountered during the pandemic. Compared to before the pandemic, women have reported increased overall stress due to their household setting, additional responsibilities, and financial concerns. Children were in school while parents would be at work, daycare and babysitting services were available and affordable, and afterschool routines were manageable prior to the pandemic. Further, researchers have had to allocate their work hours toward more clinical or administrative work rather than their ongoing projects, and grant money and proposal deadlines have become difficult to reach. In addition, prior to the pandemic, grant proposals and submission were underway, but as seen in our results, productivity and funding became a major concern, contributing to higher stress and anxiety levels [5,7]. Interestingly, there were negative associations seen between anxiety levels and clinical effort in the entire group and more so in the subgroup of scientists. This may perhaps be due to lower expectations for productivity among those with more clinical effort or due to availability of another career track if research efforts are unsuccessful. These effects could hinder any long-term progression of women’s trajectory in academic medical research.

In spite of the new stressors associated with the pandemic, our results also show some important positive findings. The majority felt supported by their institution or their department. In terms of coping mechanisms, 88.3% did have someone to speak with regarding their concerns, although the individuals were primarily outside the work setting. The majority did not experience financial difficulties, salary cuts, or furloughs, and the age of children did not correlate to stress and anxiety levels as there were no significant differences seen in levels of stress and anxiety when reporting school-age children in the household, responsibility level, and/or being in charge of e-learning.

We can learn from previous pandemics and recessions where the impacts on women in healthcare sciences research resulted in pay gaps increasing by 3.7% for women in addition to a decline in leadership roles as women are overlooked due to a redirection to outside obligations during times of crises [10,11]. Our data illustrate a need for attentive care of working women in healthcare sciences research, who feel undersupported in their respective field of work, especially when compared to their male counterparts or colleagues without children [12–14] and/or additional obligations outside of work. Academic and healthcare institutions and funding organizations should consider providing strategic support, equitable extensions, and allocated funding to those unfairly impacted by crises. This pandemic has shed a light on the policies that may need to be put in place to better prepare and support women in healthcare sciences research, whether it be extensions on grant deadlines, increased administrative support, and alternatives to delayed research with added clinical responsibilities or administrative tasks that have needed to be taken on by these women [10,11].

It is also important to address the outliers of this study that were brought to light as a result of the COVID-19 pandemic. A few women voiced concerns in regard to issues on social and racial injustices (data not shown) coinciding with the onset of the pandemic. Institutions should want to look into providing those who feel underrepresented and unsupported in the workplace with an uplifting support network. As participants mentioned these issues to be affecting their stress levels on both a professional and personal level, institutions should be aware of the emotional impacts social and racial injustice have on their faculty and implement effective means of support; be it by providing outlets for open discussion and a communitive environment to colleagues with contacts and resources to reach out to if ever in need.

Women who feel misunderstood by their colleagues, either because they do not have children, are males or can simply not relate to their situation, also need to be recognized. Women in our study are stating that they are uncomfortable or afraid to speak up about their concerns. Institutions may want to address these issues by implementing social support systems and routine supervisor-colleague sessions. Women should have a place where they are able to bring about concerns without judgment or fear of potential career impacts. Trainings should be implemented in the workplace to allow for unbiased, active discussions on how to best address the concerns of women in academic appointments. One suggestion would be to implement a peer group within the workplace so that women can connect with those within the institution that they can most relate to, and if it is not available, there should be administrative efforts made to support these women and allow them to address concerns openly and freely.

Overall, it is critical to take into consideration the impact of the COVID-19 pandemic on women in healthcare sciences research. By opening up the conversation on these topics of concern, effective change in the workplace on both an institutional and systematic level can be made. Allocation of and access to resources for those struggling with abrupt work-life alterations should be made available at all times. Redefining measures of scholarly productivity and providing flexible policies should be implemented for early-career women in healthcare sciences research. Further, efforts should be made to optimize the work environment (given it is the primary place of productivity) as well as provide childcare support through institutional daycare centers.

This study is not without limitations. This survey was conducted early during the pandemic, but after the shutdown mandate was removed in most states. Thus, the sentiments and stressors may have changed over time. In addition, appropriate validation of the survey questions had not been performed prior to its dissemination; however, this is a common practice in an emergency or worldwide pandemic, where capturing the emotions in a timely manner is more pressing. Further, this study was piloted and tested among the research team. This study also assessed marital status with childcare and clinical responsibility allocations. While there was a majority correlation, it should be noted that our respondent population was majority married with children, thus impacting the generalizability of our results. We did not explore further the specifics of caring for other family members, whether seniors or those with special needs, except in that case of children where we asked about the age of children and e-learning responsibilities. Identifying stressors such as family members with special needs or seniors in the household does help in further understanding their possible contribution to stress and anxiety among those individuals. The data collected also consisted of majority White women, so findings cannot be generalized, and significant correlations could not be made. In addition, as only women were surveyed, adequate correlations to men’s perceptions of the COVID-19 impact were not collected. While many women stated their concerns of being compared to their male colleagues or unfair advantages, we cannot say for certain that men do not also hold similar concerns, or that it solely applies to women. Men may also have financial concerns, time management, and family obligations or childcare responsibilities that were not noted in this study. In addition, we could not identify a normative comparator group to compare levels of stress and anxiety to. This is because every field is highly specialized, and results from one group may not be applicable to others. Yet, it is still important to emphasize the pandemic concerns mentioned in this study to better establish policies and support networks that would benefit and create a level playing field for all individuals involved. This study also measured several aspects and potential variables contributing to stress and anxiety and surveyed a good representative number of women engaged in health sciences research across the country.

This study focused on early- to mid-career women in healthcare sciences research. It would be interesting to look further into those in later academic standings, to see if their opinions on the pandemic – whether their standing in respective research appointments and their perception of the impact on their careers – are comparable to the majority assessed in this study. As this study did not survey men, it would be valuable to distribute and compare their opinions and perceptions of COVID-19 on their career journeys as well. Lastly, by expanding this survey over a longer time period to more age groups, ethnicities, and marital statuses, a more diverse population would be beneficial in assessing whether or not there were differences across groups. These future modifications would add to the generalizability and significance of our study correlations and bring about further necessary conversations.

Overall, this study indicated there were greater levels of stress, worry, and challenges of work-life balance compared to before pandemic. This study also provides insight into helping those individuals during any future pandemics or emergency situations. It appears that our results affirm that institutions play a central role in mitigating stress and anxiety due to financial concerns, and perhaps greater attention can be focused on the well-being and resilience of women in healthcare sciences research so that challenges specific to the research, productivity, and the healthcare environment can be addressed.

## Supplementary material

For supplementary material accompanying this paper visit https://doi.org/10.1017/cts.2022.417. click here to view supplementary material
