# Supplementary material for: Emotional distress, stress, anxiety, and the impact of the COVID-19 pandemic on early- to mid-career women in healthcare sciences research
Source: J Clin Transl Sci. 2022 Jun 13;6(1):e93. doi: 10.1017/cts.2022.417 (PMC9393575; doi:10.1017/cts.2022.417)
Supplement: Supplementary file 1 [file ctssup.zip › S2059866122004174sup001.pdf]

Block 15

Dear Participant,

We would like to invite you to complete the following research survey measuring the Emotional Distress, Stress and the Impact of the COVID-19 Pandemic on Early Career Women in Healthcare Sciences Research. This research is completely voluntary and you may stop at any time.

We are seeking study participants who meet the following criteria:
1) Health Sciences/Biomedical Researcher
2) Live in the United States
3) Can read and understand English.
4) Women working in research (at least 15% research effort)
5) Early career (fellows to associate professor level).

The online survey takes no more than 15-20 minutes and includes questions about you, your background, the impact COVID-19 has had on your professional and home life. There is no compensation for participation in this study.

There are minimal risks you should be aware of. We anticipate three potential minimal risks (likelihood of occurrence is rare for each): 1) becoming upset from the questions or testing; 2) fatigue (physical or mental) during administration of questions; and 3)

loss of confidentiality.

This study is being conducted by a group of investigators at Indiana University and lead by Dr. Heba Ismail; it has been approved by the University IRB (IRB# 2006034715). You may contact Dr. Ismail at heismail@iu.edu

By participating in this study, you confirm that you understand the risks and benefits of this research study and agree that you meet the above criteria.

Preliminary Questions

Are you:

- ◯ Male
- ◯ Female
- ◯ Trans Male or Trans Man
- ◯ Trans Female or Trans Woman
- ◯ Gender Non-conforming or Gender Queer
- ◯ Other
- ◯ Prefer not to answer

Preliminary Questions 2

Are you engaged in health sciences/biomedical research (at least 15% of your time is spent doing research)?

- ◯ Yes
- ◯ No

Basic Career Questions

What is your highest degree? - Select all that apply

- ☐ MD
- ☐ PhD
- ☐ MSc
- ☐ MPH
- ☐ [ ] Other: Please specify

How long have you been conducting research?

- ◯ < 6 months
- ◯ 1-5 years
- ◯ 5-10 years
- ◯ 15-20 years
- ◯ > 20 years

What is your current position? - Select all that apply

- ☐ Postdoctoral Fellow
- ☐ Clinical Fellow
- ☐ Assistant Professor
- ☐ Associate Professor
- ☐ Professor
- ☐ Administrative Position
- ☐ ____________ Other: Please specify

Are you a:

- ○ Scientist
- ○ Physician Scientist

What percent effort do you do clinical/teaching/administrative tasks? - Please type a number.

Clinical	0
Teaching	0
Administrative	0
Research	0

Total 0

How long have you been in your current position?

- ◯ < 6 Months
- ◯ 1-5 Years
- ◯ 5-10 Years
- ◯ 15-20 Years
- ◯ > 20 Years

Where do you work? - Select all that apply

- ☐ Large Private University
- ☐ Small Private University
- ☐ Large Public University
- ☐ State University
- ☐ Community College
- ☐ ________ Other: Please specify

What is your department or speciality?

- ◯ Internal Medicine
- ◯ Pediatrics

- ◯ Nursing
- ◯ Surgery
- ◯ Dentistry
- ◯ Public Health
- ◯ Pharmacy
- ◯ Behavioral Medicine
- ◯ ____ Other: Please specify

COVID-19 and Research Questions

Due to the COVID-19 Pandemic, please indicate weather the following has occurred: - Select all that apply

- ☐ My Lab is Closed
- ☐ My Clinical Research is on Hold
- ☐ My Animal Research is on Hold
- ☐ ____ Other: Please explain

Due to the COVID-19 Pandemic, I have taken on work responsibilities usually assumed by others (such as research assistants, project managers, research coordinators, nurses, or administration).

- ◯ Yes

◯ No

If yes, to what extent have you taken on more responsibilities?

Not At All	A Little	Somewhat	Quite a Bit	Very Much
◯	◯	◯	◯	◯

COVDI-19 Support and Concerns

For the following statements, please check the response that best describes your level of support during the COVID-19 Pandemic. I have felt support by:

	Not At All	A Little	Somewhat	Quite a Bit	Very Much
My institution	◯	◯	◯	◯	◯
My department	◯	◯	◯	◯	◯

For the following statements, please check the response that best describes your level of concern on the effect of the COVID-19 Pandemic. I am concerned about:

	Not At All	A Little	Somewhat	Quite a Bit	Very Much
Reaching my research productivity goals	◯	◯	◯	◯	◯

	Not At All	A Little	Somewhat	Quite a Bit	Very Much
Academic promotion, tenure, graduation from training, or job search	○	○	○	○	○
Current funding, grants, and/or future funding	○	○	○	○	○
Salary cuts or furloughs	○	○	○	○	○

What are your worries about the COVID-19 Pandemic and your professional career and goals?

What are your biggest professional concerns?

How has the Pandemic impacted your professional life and career?

COVID-19 Concerns and Communication

For the following question, please select the choice that best corresponds to your views:

	Not at All	A Little	Somewhat	Quite a Bit	Very Much
I communicate a lot with other professionals in the situation	◯	◯	◯	◯	◯

Do you have someone to talk to about your concerns? What is their relationship to you and how often do you talk with them?

COVID-19 and Fincances

Have there been any salary cuts or furloughs to you, your staff, or anyone in your research team?

- ◯ Yes
- ◯ No

Have you been asked to redeploy to clinical duties or provide information on willingness to redeploy?

- ◯ Yes
- ◯ No
- ◯ N/A

Have you lost your job due to COVID-19?

- ◯ Yes
- ◯ No

Have you experienced financial difficulty related to COVID-19?

- ◯ Yes
- ◯ No

Please indicate the range of your total annual family income:

- ◯ Less than $50,000
- ◯ $50,000 - $75,000
- ◯ $75,000 - $99,000
- ◯ $99,000 - $125,000
- ◯ $125,000 - $150,000
- ◯ $150,000 - $175,000
- ◯ $175,000 - $200,000
- ◯ More than $200,000
- ◯ ________ Other: Please specify

Household Information

What is your current marital/family status?

- Married without Children
- Married with Children
- Divorced without Children
- Divorced with Children, Shared Custody
- Divorced with Children, Full Custody
- Widowed without Children
- Widowed with Children
- Single without Children
- Single with Children Part Time
- Single with Children Full Time

Who lives in your household? - Select all that apply

- Adults
- Children
- Seniors (> 65 Years Old)
- Seniors with Special Needs

If you have children in your household, select all that apply:

- Children < 5 Years Old

- ☐ Children Between 5 and 12 Years
- ☐ Teenagers < 18 Years Old
- ☐ Children with Special Needs

If you have children, are/were they receiving e-learning at home during the Pandemic?

- ◯ Yes
- ◯ No

Are/were you in charge of schooling and e-learning?

- ◯ Yes, Full-time
- ◯ Yes, Part-time
- ◯ No

Due to the effects of the COVID-19 Pandemic, the responsibility of childcare (or adult/senior care) has now:

- ◯ Fallen mostly on me
- ◯ Is currently equally shared with others
- ◯ Is currently unequally shared with others (I do most of it)
- ◯ Is currently unequally share with others (others do most of it)

◯ N/A

COVID-19 and Productivity

Due to the effects of the COVID-19 Pandemic: - Select all that apply

- ☐ My research work hours are reduced
- ☐ My research productivity has been reduced
- ☐ My research work hours increased
- ☐ My research productivity has increased
- ☐ ________ Other: Please specify

If research productivity has been reduced, is this due to: - Select all that apply

- ☐ Research work hours are reduced
- ☐ Stress
- ☐ Lack of focus/distraction
- ☐ Depression
- ☐ Lack of motivation
- ☐ External factors
- ☐ Conditions at home
- ☐ ________ Other: Please specify

COVID-19 Diagnosis and Research

Have you or anyone you know been diagnosed with COVID-19?

- Yes
- No

The person I know that has been diagnosed with COVID-19 is: - Select all that apply

- Me
- A household member
- A close relative
- A remote relative
- A close friend
- An acquaintance
- Other: Please specify

Are you currently caring for any patients who have been diagnosed with COVID-19?

- Yes
- No

Have you spent any time seeking COVID-19 related health information for yourself or a family member? - Select all that apply

- ☐ Yes, for myself
- ☐ Yes, for a family member
- ☐ No

Have you received any COVID-19 related health information that you did not try to find (i.e. passively for example: by email, on social media, on TV, radio, or other source)?

- ◯ Yes
- ◯ No

COVID-19 and feelings

For the following questions, please select the choice that best corresponds to your views:

	Not at All	A Little	Somewhat	Quite a Bit	Very Much
How much does COVID-19 affect your life?	○	○	○	○	○
Does COVID-19 make you feel angry?	○	○	○	○	○
Does COVID-19 make you feel scared?	○	○	○	○	○
Does COVID-19 make you feel upset?	○	○	○	○	○
Does COVID-19 make you feel depressed?	○	○	○	○	○
Does COVID-19 make you feel anxious?	○	○	○	○	○

For the following questions, please select the choice that best corresponds to your views:

	Much Less Than Before	Less Than Before	The Same	More Than Before	Much More Than Before
How would you compare your stress level during the COVID-19 pandemic to your stress level before the pandemic started?	○	○	○	○	○

	Much Less Than Before	Less Than Before	The Same	More Than Before	Much More Than Before
How would you compare your anxiety level during the COVID-19 pandemic to your stress level before the pandemic started?	◯	◯	◯	◯	◯

COVID-19 and Self - Efficacy

We would like to know how confident you are in doing certain activities during the COVID-19 pandemic. For each of the following questions, please select the choice that corresponds to your confidence that you can do the tasks regularly at the present time.

	Not at All	A Little	Somewhat	Quite a Bit	Very Much	N/A
How confident are you that you can take care of yourself with the added burden of social distancing?	◯	◯	◯	◯	◯	◯
How much does emotional distress caused by COVID-19 interfere with taking care of yourself?	◯	◯	◯	◯	◯	◯

	Not at All	A Little	Somewhat	Quite a Bit	Very Much	N/A
How much does emotional distress caused by COVID-19 interfere with the management of household members with special needs?	◯	◯	◯	◯	◯	◯
How confident do you feel that you can manage your household members with special needs at home?	◯	◯	◯	◯	◯	◯

COVID-19 and Stress

We would like to ask about your feeling and thoughts during the last month. For the following cases indicate how often you felt or thought a certain way. In the last month, how often have you:

	Never	Almost Never	Sometimes	Fairly Often	Very Often
Been upset because of something that happened unexpectedly?	◯	◯	◯	◯	◯
Felt nervous and stressed	◯	◯	◯	◯	◯
Felt that you were unable to control the important things in your life?	◯	◯	◯	◯	◯

	Never	Almost Never	Sometimes	Fairly Often	Very Often
Felt confident about your ability to handle personal problems?	○	○	○	○	○
Felt that things were going your way?	○	○	○	○	○
Found that you could not cope with all the things that you had to do?	○	○	○	○	○
Been able to control irritations in your life?	○	○	○	○	○
Felt that you were on top of things?	○	○	○	○	○
Been angered because of things that were outside of your control?	○	○	○	○	○
Felt difficulties were piling up so high that you could not overcome them?	○	○	○	○	○

How do you deal with the stress created by the COVID-19 Pandemic?

Emotional Distress and Anxiety

For the following questions, please select the choice that best corresponds to your views in the past 7 days:

	Never	Rarely	Sometimes	Often	Always
I felt fearful	○	○	○	○	○
I found it hard to focus on anything other than my anxiety	○	○	○	○	○
My worries overwhelmed me	○	○	○	○	○
I felt uneasy	○	○	○	○	○
I felt nervous	○	○	○	○	○
I felt like I needed help for my anxiety	○	○	○	○	○
I felt anxious	○	○	○	○	○
I felt tense	○	○	○	○	○

What has been the most challenging part of the COVID-19 Pandemic and managing your professional or personal life?

Demographics

How old are you?

	0	10	20	30	40	50	60	70	80	90	100
Move the slider to select your age											

What is your race/ethnicity? - Select all that apply

- ☐ American Indian/Alaska Native
- ☐ Asian
- ☐ Black/African American

- [ ] Caucasian
- [ ] Hispanic/Latino
- [ ] Native Hawaiian/Pacific Islander
- [ ] ________ Other: Please specify

Powered by Qualtrics
