# Supplementary material for: Emotional distress, stress, anxiety, and the impact of the COVID-19 pandemic on early- to mid-career women in healthcare sciences research
Source: J Clin Transl Sci. 2022 Jun 13;6(1):e93. doi: 10.1017/cts.2022.417 (PMC9393575; doi:10.1017/cts.2022.417)
Supplement: Supplementary file 1 [file ctssup.zip › S2059866122004174sup002.docx]

Supplemental Table 1: COVID-19 feelings and associations with levels of stress and anxiety (p and X^2^ listed only for significant associations):

Supplemental Table 2: Self-Efficacy and measures of Stress and Anxiety.

Supplemental table 3: Open-ended question responses categorized by worries related to the pandemic and professional careers and goals:

| Worries about the COVID-19 Pandemic and professional career and goals | N | % |
| --- | --- | --- |
| Productivity loss | 78 | 51.7 |
| Job concerns and Future | 52 | 34.4 |
| Additional Stressors (childcare, other responsibilities) | 32 | 21.2 |
| Work Dynamic | 7 | 4.6 |
| Financial Pressures | 34 | 22.5 |
| None | 2 | 1.3 |
